# Supplementary material for: MCMV Dissemination from Latently-Infected Allografts Following Transplantation into Pre-Tolerized Recipients
Source: Pathogens. 2020 Jul 26;9(8):607. doi: 10.3390/pathogens9080607 (PMC7460028; doi:10.3390/pathogens9080607)
Supplement: Supplementary file 1 [file pathogens-09-00607-s001.pdf]

Parameter	Value
STZ	170 mg/kg
ECDIsp Dose	$1x10^8$ cells
Approximate Islets Transplanted	300 islets
Blood Glucose of all mice prior to islet transplant	Greater than 250 mg/dl

Supplemental Figure 1. Treatment parameters for islet transplants. Parameters related to diabetes induction, islet transplant mass, and ECDIsp cellular dosage.

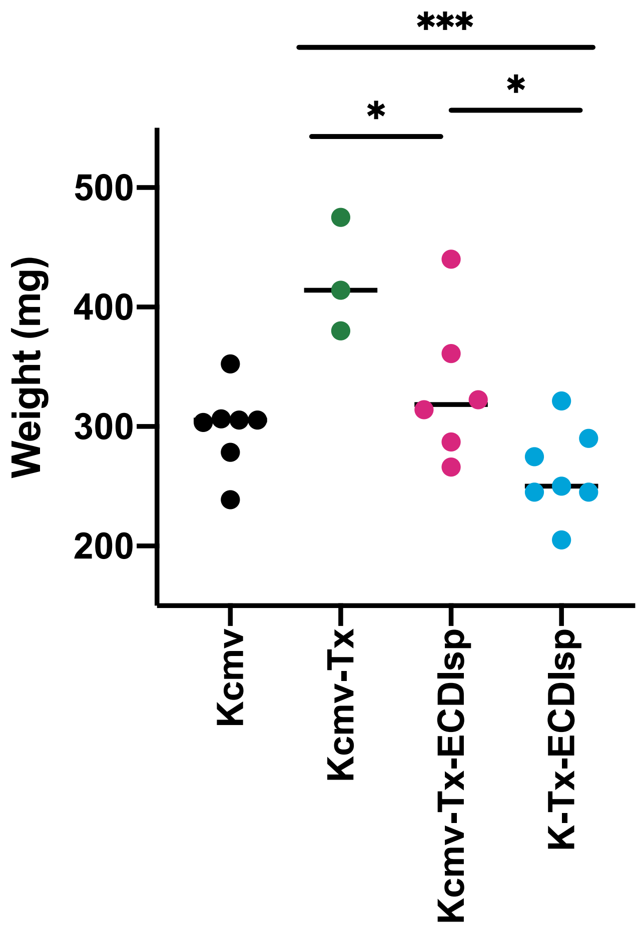

Supplemental Figure 2. Weights of kidney grafts from indicated groups at 30-35 days post transplant

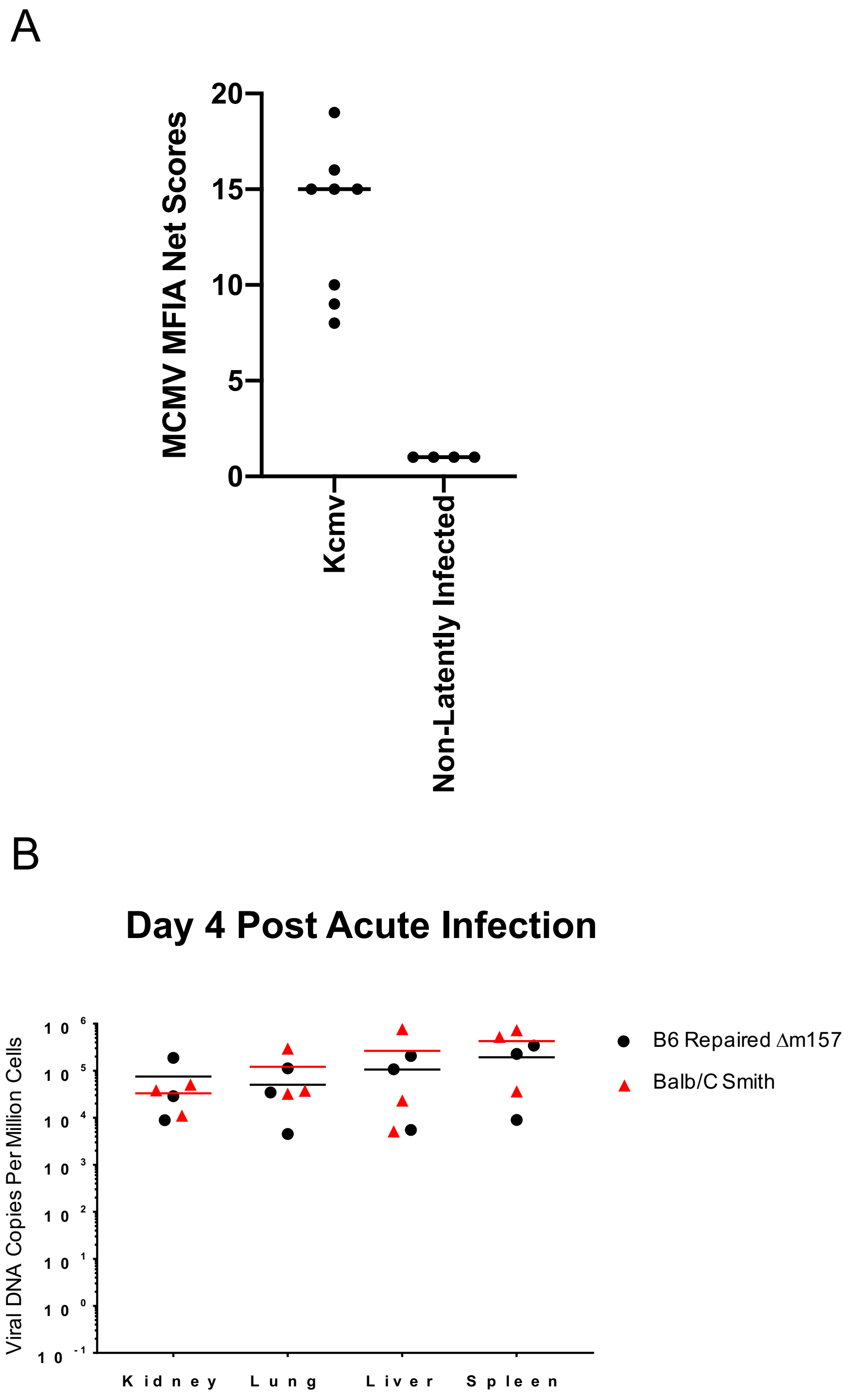

Supplemental Figure 3. MCMV serology confirmation. (A) MCMV Multiplexed Fluorometric Immune Assay (MFIA) was conducted on serum of latently infected and non-latently infected B6 mice by Charles River Research. Latency was confirmed by MCMV MFIA Net Scores: 1 is negative, 2 is equivocal and 3 or higher is positive. n=4-8 mice per group. (B) Viral DNA copies for *C57BL/6* mice acutely infected with repaired Δm157 virus or *BALB/c* mice infected with MCMV Smith virus. Organs were collected 4 days post infection and *MCMV* immediate early gene (*IE1)* copy numbers determined.
